# Intramuscular administration of autologous total immunoglobulin G induces immunomodulatory effects on T cells in healthy human subjects: An open-labeled prospective single-arm trial

**DOI:** 10.1097/MD.0000000000029486

**Published:** 2022-06-03

**Authors:** Byul Kwon, Seung-Jung Yang, Su-Mi Cho, Myoung-Eun Kim, Dong-Ho Nahm

**Affiliations:** Department of Allergy and Clinical Immunology, Ajou University School of Medicine, Suwon, Gyeonggi-do, Korea.

**Keywords:** clinical trial, human subjects, immunoglobulin, immunomodulation, T cell

## Abstract

**Background:**

We hypothesized that intramuscular administration of autologous total immunoglobulin G (IgG) could induce an immunomodulatory effect in human subjects. In our previous studies, we showed that intramuscular administration of autologous total IgG could induce significant clinical improvements and increases of the serum levels of interleukin-10 (IL-10) and interferon-gamma (IFN-γ) in patients with atopic dermatitis.

**Objective:**

To investigate the mechanism of immunomodulation induced by intramuscular administration of autologous total IgG, we evaluated changes in T cells before and after intramuscular administrations of autologous total IgG in this study.

**Methods:**

Thirteen healthy adults received 8 intramuscular injections of 50 mg autologous total IgG for 4 weeks (from week 0 to week 4). The percentages of IL-10- or IFN-γ-producing peripheral blood T cells, as well as serum levels of IL-10, IFN-γ, and immunoglobulins, were measured at baseline (week 0) and at weeks 4, 8, and 12.

**Results:**

The percentage of IL-10-producing CD4^+^ T cells was significantly increased at weeks 8 and 12 compared to baseline (*P* < .05), while the percentage of IFN-γ-producing CD3^+^ T cells was significantly increased at week 12 compared to baseline (*P* < .05). There were no significant differences in the serum levels of IL-10, IFN-γ, and immunoglobulins before and after intramuscular administration of autologous total IgG (*P* > .05). No serious adverse events were observed.

**Conclusion:**

Intramuscular administration of autologous total IgG induced immunomodulatory effects on T cells in healthy human subjects. This simple intervention could be a safe, effective, and economical T cell immunomodulation method for human subjects (NCT03695757).

## 1 Introduction

Intravenous administration of polyvalent human immunoglobulin G (IgG) purified from the plasma pool of multiple healthy human blood donors has been used for the treatment of patients with primary immunodeficiency diseases associated with decreased production of immunoglobulin.^[1]^ Due to its immunomodulatory effects, this therapy has also been used for the treatment of various autoimmune and allergic diseases.^[2,3]^ However, the mechanism of immunomodulation induced by polyvalent IgG in human immune diseases remains elusive, even though 4 decades have passed since the first clinical trial in children with idiopathic thrombocytopenia in 1981.^[2–5]^

The current evidences suggest that polyvalent IgG modulates the function of immune cells, including dendritic cells, neutrophils, monocytes, macrophages, T cells, and B cells.^[6]^ Previous studies have showed that a stimulation of interleukin-10 (IL-10)-producing CD4^+^ regulatory T cell is critical for immunomodulatory and anti-inflammatory effects of polyvalent IgG.^[7–9]^ Natural IgG antibodies to the antigen binding sites (idiotypes) of pathogenic antibodies (IgG autoantibodies or IgE antibodies) have been suggested as the main therapeutic components of polyvalent IgG exerting immunomodulatory effects by neutralizing pathogenic antibodies.^[10–12]^

The idiotype-anti-idiotypic immune response has been suggested to contribute to the development and maintenance of immune homeostasis (immune tolerance) in healthy human subjects.^[13–15]^ We hypothesized that active induction of anti-idiotypic immune response to pathogenic antibodies by intramuscular administration of autologous total immunoglobulin containing pathogenic antibodies could induce an immunomodulatory effect and provide a clinical improvement in patients with autoimmune and allergic diseases.^[16]^

The concept of this study, an induction of immunomodulatory effect by intramuscular administration of autologous total IgG in healthy human subjects, originated from our understanding of the therapeutic mechanism of autologous blood therapy.^[17,18]^ Autologous blood therapy or autologous serum therapy (also called autohemotherapy or autoserum therapy, respectively) involves repeated administrations of a small amount (1–5 mL) of autologous blood or serum to the same subject by intramuscular injections, immediately after venous blood sampling.^[17–21]^ These have been practiced as common alternative and complementary medical modalities to treat various immune diseases for more than 100 years since the first report in 1913.^[19–22]^ Randomized, double-blind, placebo-controlled studies have demonstrated favorable clinical efficacies of autologous blood therapy and autologous serum therapy in patients with atopic dermatitis and chronic urticaria, respectively.^[19,20]^ However, the therapeutic component of blood producing the clinical efficacy of autologous blood therapy and the underlying therapeutic mechanism have not been identified. We hypothesize that the blood component responsible for the therapeutic efficacy of autologous blood therapy is an autologous total immunoglobulin, including pathogenic antibodies, and that the therapeutic mechanism is an anti-idiotypic immunomodulation induced by the intramuscular administration of autologous total immunoglobulin.^[16]^

To prove the concept, we evaluated the clinical efficacy, safety, and immunomodulatory effect of intramuscular administration of autologous total IgG in 20 adult patients with severe atopic dermatitis as an open-labeled prospective single-arm pilot clinical trial.^[16,23–25]^ In that study, intramuscular administration of 50 mg autologous total IgG twice a week for 4 weeks significantly decreased the clinical severity scores of atopic dermatitis and serum total IgE levels, and significantly increased serum levels of IL-10 and interferon-gamma (IFN-γ) at weeks 4, 8, and 12 compared to baseline (week 0) without serious adverse events.^[16,23–25]^ In a subsequent randomized, double-blind, placebo-controlled study, we demonstrated that 8 weekly intramuscular administrations of 50 mg autologous total IgG over a 7-week period could induce significant clinical improvements and increases of serum levels of IL-10 and IFN-γ at weeks 4, 8, 12, and 16 compared to baseline in 51 adolescent and adult patients with moderate-to-severe atopic dermatitis without serious adverse events.^[26]^ These results showed that intramuscular administration of autologous total IgG could provide a systemic immunomodulatory effect in patients with atopic dermatitis.

To further evaluate the mechanism of immunomodulation induced by intramuscular administration of autologous total IgG, we evaluated changes in T cell before and after intramuscular administration of autologous total IgG in 13 healthy human subjects.

## 2 Methods

### 2.1 Study design

This study was conducted at a single academic center (Ajou University Hospital, Suwon, Korea) as an open-labeled prospective single-arm pilot clinical trial. Study subjects were recruited from November 2018 to May 2019. This study was conducted in compliance with the guidelines for Good Clinical Practice and the Declaration of Helsinki with the approval from the institutional review board of Ajou University Hospital. All study participants provided written informed consent. This trial is registered with ClinicalTrials.gov (NCT 03695757).

### 2.2 Study participants

Thirteen healthy adult human subjects (age ≥19 years) without underlying disease, and compatible with the criteria for autologous blood donation (hemoglobin level ≥11.0 g/dL and body weight ≥40 kg)^[27]^ participated in this study (Table 1). Exclusion criteria for this study were as follows: pregnancy, lactation, alcohol addiction, and systemic diseases. The health status of the subjects was determined based on medical history, physical examination, electrocardiogram, chest X-ray, and laboratory tests (complete blood cell count, routine chemistry tests for liver and kidney functions, and serological tests for human immunodeficiency virus, and hepatitis B and C viruses).

**Table 1:** Baseline characteristics of the 13 healthy human subjects who participated in this study.

Age, mean (SD), yr	24.0 (4.1)
Sex, n (%)	
Man	8 (61.5%)
Women	5 (38.5%)
Body weight (kg), mean (SD)	61.3 ± 8.4
Body mass index, mean (SD)	21.6 ± 1.7

Data are presented as numbers with percentages or means ± standard deviation (SD).

### 2.3 Preparation of autologous total IgG

**Figure 1:**
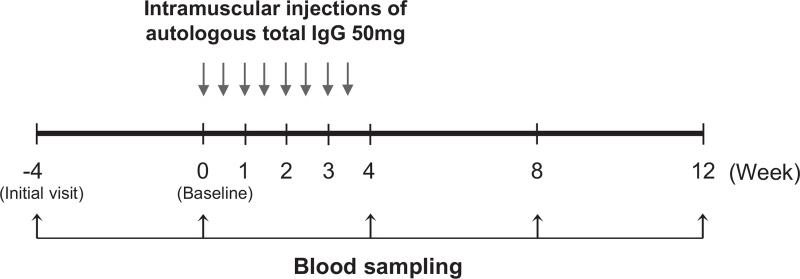
The study design.

Autologous venous blood (400 mL) was collected from each participant using a double blood bag containing anticoagulant (Green Cross PBM, Seoul, Korea) at the initial visit (week −4) (see the study design in Fig. 1). Autologous plasma (approximately 200 mL) was separated from the autologous venous blood by centrifugation. Approximately 500 mg of autologous total IgG (purity ≥97%) was aseptically purified from the autologous plasma by affinity chromatography using Protein A beads, as previously described.^[16,28]^ The solution containing the purified autologous total IgG was tested for bacterial contamination using an endotoxin assay kit (Associates of Cape Cod Inc., Falmouth, MA). The autologous total IgG solution was transferred into sterile glass vials in 50-mg aliquots and stored at −20°C.

### 2.4 Intramuscular administration of autologous total IgG and venous blood sampling

The frozen autologous total IgG solution was thawed at room temperature for intramuscular injection. Study participants received 8 intramuscular injections of 50 mg autologous total IgG twice a week for 4 weeks (from week 0 to week 4) and then were followed until week 12 (Fig. 1). Venous blood was obtained at the initial visit (week −4), at baseline (week 0), and at weeks 4, 8, and 12. Serum samples were stored at −20 °C.

### 2.5 Flow cytometric analysis of IL-10- and IFN-γ-producing T cells

Peripheral blood mononuclear cells were isolated from venous blood by density gradient centrifugation using Cell Preparation Tubes (BD Biosciences, San Jose, CA) at baseline (week 0) and weeks 4, 8, and 12. Flow cytometric analysis was performed as previously described.^[29]^ Peripheral blood mononuclear cells were incubated in RPMI 1640 medium (Corning, Manassas, VA) supplemented with 10% heat-inactivated fetal bovine serum, 1% penicillin/streptomycin, 2 mM l-glutamine, and Cell Stimulation Cocktail (plus protein transport inhibitors) containing phorbol 12-myristate 13-acetate (PMA), ionomycin, brefeldin A, and monensin (eBioscience, San Diego, CA) for 20 hours at 37°C with 5% CO_2_. Next, cells were incubated with PerCP-Cyanine5.5-labeled anti-CD3 (SK7, eBioscience), FITC-labeled anti-CD4 (RPA-T4, eBioscience), PE-labeled anti-CD4 (RPA-T4, eBioscience), PE-Cyanine7-labeled anti-CD8 (SK1, eBioscience), APC-labeled anti-CD25 (BC96, eBioscience), and PE-Cyanine7-labeled anti-CD19 (HIB19, eBioscience) antibodies for 30 minutes at 4 °C. For intracellular staining, cells were incubated with fixation/permeabilization buffer (eBioscience) for 30 minutes at 4 °C, followed by incubation with PE-labeled anti-IL-10 (JES3-9D7, eBioscience) and FITC-labeled anti-IFN-γ (4S.B3, eBioscience) antibodies. Flow cytometric analysis was performed using a BD FACSAria III flow cytometer (BD Biosciences). Data were analyzed using the FACSDiva software version 7.0 (BD Biosciences).

### 2.6 Serum levels of IL-10 and IFN-γ

Serum levels of IL-10 and IFN-γ were measured using enzyme-linked immunosorbent assay (ELISA) monoclonal antibody kits and standards (BD Biosciences) according to the manufacturer's instructions.

### 2.7 Serum concentrations of immunoglobulin

Serum concentrations of IgG, IgA, and IgM were measured by a turbidimetric immunoassay using a COBAS INTEGRA analyzer (F. Hoffmann-La Roche, Basel, Switzerland). The serum total IgE level was measured using the ImmunoCAP assay (Thermo Fisher Scientific, Waltham, MA).

### 2.8 Serum levels of IgG antibodies to environmental antigens and autoantigens

Serum levels of IgG antibodies to pneumococcal capsular polysaccharide antigens (GlaxoSmithKline Biologicals, Rixensart, Belgium), influenza antigens (GlaxoSmithKline, Dresden, Germany), house dust mite antigens (whole body extract of *Dermatophagoides farinae*, HollisterStier, Spokane, WA), and bovine thymus extract (Immunovision, Springdale, AR), as extractable nuclear antigens, were measured using ELISA as previously described.^[30–33]^ Serum levels of IgG antibodies to those antigens were expressed in arbitrary units using serially diluted high-titer serum samples as standards.

### 2.9 Other laboratory parameters

Complete blood cell counts, including erythrocyte, platelet, and total and differential leukocyte (neutrophil, lymphocyte, monocyte, eosinophil, and basophil) counts were assessed using an automated hematology analyzer (Coulter Counter STKS, Beckman Coulter, Fullerton, CA). Routine chemistry tests, including liver and kidney function tests were performed using a Cobas c702 analyzer (Roche Diagnostics, Basel, Switzerland).

### 2.10 Statistical analysis

Data are presented as the mean ± standard error of the mean. Statistical significance of the differences in values before and after intramuscular administration of autologous total IgG was analyzed using the Wilcoxon signed-rank test. Bonferroni correction was applied to compensate Type I statistical error developed due to multiple comparisons. A corrected *P* value was obtained by Bonferroni correction. The Bonferroni corrected *P* value (calculated by multiplying the original *P* value with the number of comparisons) <.05 was considered statistically significant.

## 3 Results

### 3.1 IL-10-and IFN-γ-producing T cells

**Figure 2:**
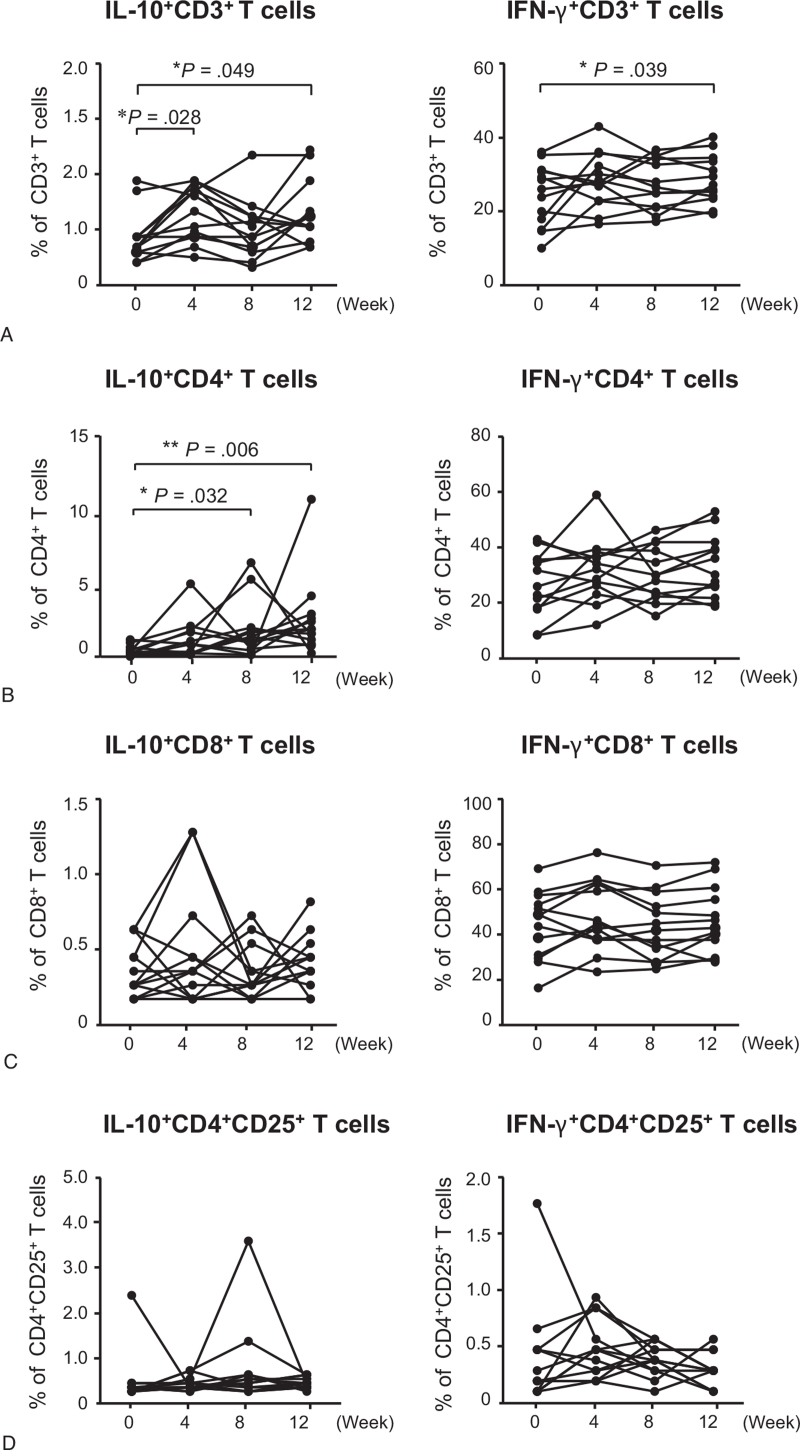
The percentages of IL-10- or IFN-γ-producing peripheral blood CD3^+^ (A), CD4^+^ (B), CD8^+^ (C), and CD4^+^CD25^+^ (D) T cells in 13 healthy human subjects who received 8 intramuscular administrations of 50 mg autologous total IgG twice a week for 4 wk (from week 0 to week 4) during the 12-wk study period. *P* values were calculated using the Wilcoxon signed-rank test with Bonferroni correction. Each symbol and connecting line in the graphs represents an individual healthy subject. ^∗^*P* < .05, ^∗∗^*P* < .01 compared to baseline.

**Figure 3:**
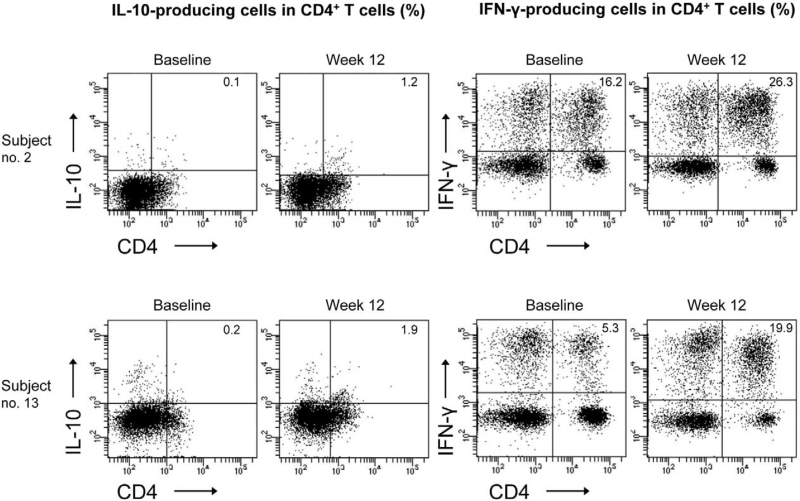
Representative flow cytometric analysis data on the percentages of IL-10- or IFN-γ-producing peripheral blood CD4^+^ T cells at baseline (week 0) and week 12 in 2 healthy human subjects who received 8 intramuscular administrations of 50 mg autologous total IgG twice a week for 4 wk (from week 0 to week 4) during the 12-wk study period.

**Figure 4:**
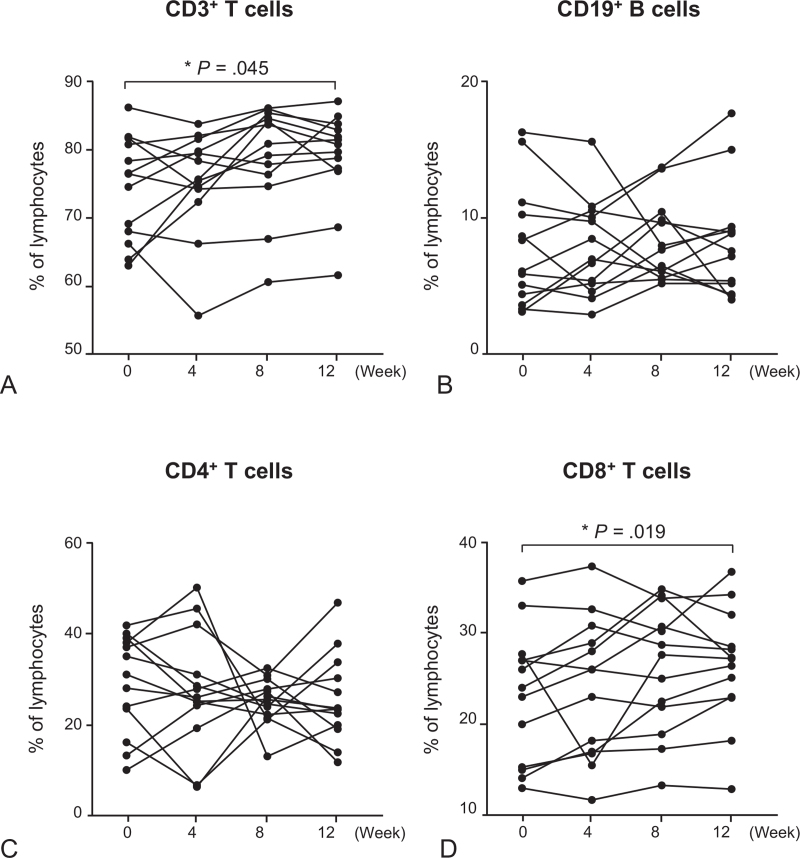
Subpopulations of peripheral blood lymphocytes cultured for 20 h with non-specific cell stimulants in 13 healthy human subjects who received 8 intramuscular administrations of 50 mg autologous total IgG twice a week for 4 wk (from week 0 to week 4) during the 12-wk study period. *P* values were calculated using Wilcoxon's signed-rank test with Bonferroni correction. Each symbol and connecting line in the graphs represents an individual healthy subject. ^∗^*P* < .05 compared to baseline.

The percentage of IL-10-producing CD3^+^ T cells was significantly increased at weeks 4 and 12 compared to baseline (*P* < .05) (Fig. 2 and see Table S1, Supplemental Digital Content, which describes the changes of percentages of IL-10- and IFN-γ-producing T cells in 13 healthy human subjects). The percentage of IL-10-producing CD4^+^ T cells was also increased at weeks 8 and 12 compared to baseline (*P* < .05) (Figs. 2 and 3). The percentage of IFN-γ-producing CD3^+^ T cells was significantly increased at week 12 compared to baseline (*P* < .05) (Fig. 2). However, there were no significant differences in the percentages of IL-10- or IFN-γ-producing CD8^+^ T cells and CD4^+^CD25^+^ T cells before and after intramuscular administration of autologous total IgG (Fig. 2). The percentages of CD3^+^ T cells and CD8^+^ T cells in lymphocytes cultured for 20 hours with non-specific cell stimulants were significantly increased at week 12 compared to baseline (*P* < .05) (Fig. 4).

### 3.2 Serum levels of IL-10 and IFN-γ

**Figure 5:**
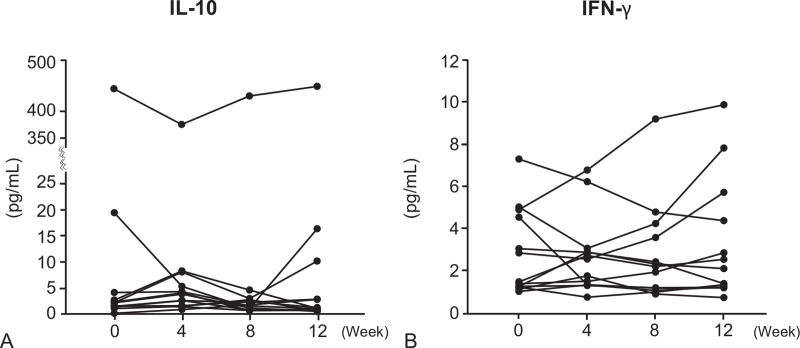
Serum levels of IL-10 and IFN-γ in 13 healthy human subjects who received 8 intramuscular administrations of 50 mg autologous total IgG twice a week for 4 wk (from week 0 to week 4) during the 12-wk study period. Each symbol and connecting line in the graphs represents an individual healthy subject.

There were no significant differences in serum levels of IL-10 and IFN-γ before and after intramuscular administration of autologous total IgG (*P* > .05) (Fig. 5).

### 3.3 Serum concentrations of immunoglobulins

There were no significant differences in serum concentrations of IgG, IgA, IgM, and IgE before and after intramuscular administration of autologous total IgG (*P* > .05) (Table 2).

**Table 2:** Changes in peripheral blood cell counts and serum concentrations of immunoglobulins in 13 healthy human subjects who received 8 intramuscular administrations of 50 mg autologous total IgG twice a week for 4 wk (from week 0 to week 4).

	Week −4 (initial visit)	Week 0 (baseline)	Week 4	Week 8	Week 12
Peripheral blood cell counts
Red blood cell (×10^6^/μL)	4.8 ± 0.1	4.6 ± 0.1	4.7 ± 0.1	4.6 ± 0.1	4.6 ± 0.1
Hemoglobin (g/dL)	14.3 ± 0.3	14.0 ± 0.4	14.1 ± 0.5	13.8 ± 0.4	13.8 ± 0.4
Platelet (×10^3^/μL)	256.2 ± 14.9	253.7 ± 15.3	254.2 ± 17.4	235.4 ± 13.5	234.6 ± 9.6
White blood cell (/μL)	5953.8 ± 359.2	5469.2 ± 363.6	5900.0 ± 359.3	5453.8 ± 327.3	5453.8 ± 282.1
Neutrophil (/μL)	3428.4 ± 293.7	3282.5 ± 307.9	3494.2 ± 267.9	3096.3 ± 274.9	3129.6 ± 233.7
Lymphocyte (/μL)	1915.6 ± 116.7	1605.3 ± 135.3	1784.5 ± 144.1	1815.4 ± 147.3	1769.8 ± 122.3
Monocyte (/μL)	433.5 ± 34.7	402.6 ± 25.1	420.4 ± 28.1	382.6 ± 33.2	382.6 ± 30.0
Eosinophil (/μL)	132.9 ± 23.8	138.4 ± 32.4	159.3 ± 28.8	117.4 ± 18.7	129.8 ± 22.8
Basophil (/μL)	43.5 ± 5.0	40.5 ± 3.4	41.6 ± 4.3	42.0 ± 4.8	42.1 ± 4.9
Immunoglobulin concentrations
IgG (mg/dL)	1182.9 ± 66.3	1154.1 ± 53.6	1152.2 ± 55.9	1149.2 ± 53.2	1149.7 ± 49.1
IgA (mg/dL)	199.8 ± 18.2	195.3 ± 19.0	199.8 ± 20.7	201.2 ± 21.3	204.1 ± 18.9
IgM (mg/dL)	108.0 ± 13.1	104.8 ± 12.8	103.9 ± 11.9	102.3 ± 13.1	102.4 ± 12.5
IgE (kU/L)	67.9 ± 26.6	62.5 ± 24.0	57.4 ± 19.5	59.1 ± 22.4	58.1 ± 21.5

Data are presented as mean ± standard error of the mean.

### 3.4 Serum levels of IgG antibodies to environmental antigens and autoantigens

**Figure 6:**
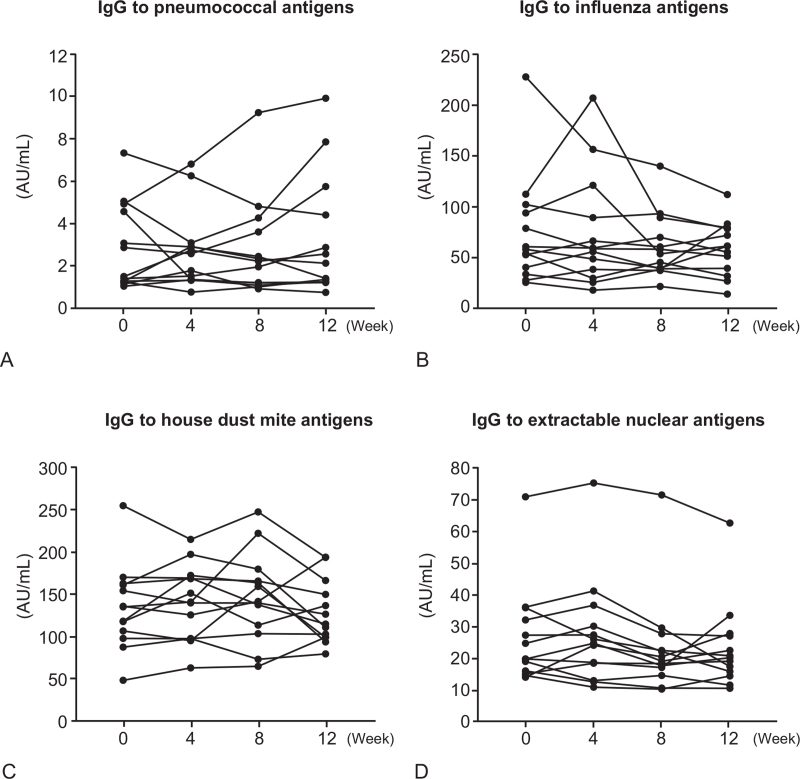
Serum levels of IgG antibodies to pneumococcal capsular polysaccharide antigens (A), influenza antigens (B), house dust mite antigens (C), and extractable nuclear antigens (D) in 13 healthy human subjects who received 8 intramuscular administrations of 50 mg autologous total IgG twice a week for 4 wk (from week 0 to week 4) during the 12-wk study period. Each symbol and connecting line in the graphs represents an individual healthy subject.

There were no significant differences in serum levels of IgG antibodies to pneumococcal capsular polysaccharide antigens, influenza antigens, house dust mite antigens, and extractable nuclear antigens before and after intramuscular administration of autologous total IgG (*P* > .05) (Fig. 6).

### 3.5 Other laboratory parameters

There were no significant differences in peripheral blood cell counts, including erythrocyte, platelet, and total and differential leukocyte (neutrophil, lymphocyte, monocyte, eosinophil, and basophil) counts at weeks 4, 8, and 12 compared to those at baseline (*P* > .05) (Table 2). In addition, there were no significant differences between the results of routine chemistry tests, including liver and kidney function tests, at baseline and week 12 (data not shown).

### 3.6 Compliance and safety of study participants

All 13 healthy human subjects who participated in this study completed all scheduled visits and procedures. Two subjects reported 2 adverse events (enterocolitis and herpes zoster) during the study period (Table 3). None of the subjects experienced serious adverse events, and no participant discontinued the intervention due to adverse events during the study period.

**Table 3:** Adverse events observed in the participants during the 12-wk study period.

Adverse events	
Subjects with 1 ≥ adverse events, n (%)	2 (15.4%)
Subjects with 1 ≥ serious adverse events, n	0
Total number of adverse events, n	2
Enterocolitis	1
Herpes zoster	1
Related adverse events, n	0
Discontinuation due to adverse events, n	0

## 4 Discussion

This study was the first clinical trial in healthy human subjects that evaluated the immunomodulatory effect and safety of intramuscular administration of autologous total IgG. Our results showed that intramuscular administration of autologous total IgG could induce significant immunomodulatory effects on T cells in healthy human subjects without serious adverse events. The immunomodulatory effects and safety of intramuscular administration of autologous total IgG in healthy human subjects suggest that this simple intervention could be a safe and effective T cell immunomodulation method functioning in human subjects regardless of their health status.

In this study, intramuscular administration of autologous total IgG significantly increased the percentage of IL-10-producing CD4^+^ T cells, as well as the percentage of IFN-γ-producing CD3^+^ T cells, in healthy human subjects. These results suggest that intramuscular administration of autologous total IgG can activate IL-10-producing CD4^+^ regulatory T cells and IFN-γ-producing T cells in healthy human subjects. In our previous study, intramuscular administration of autologous total IgG significantly increased serum IL-10 and IFN-γ levels in patients with atopic dermatitis^[26]^; however, there were no significant differences in the serum levels of IL-10 or IFN-γ before and after intramuscular administration of autologous total IgG in healthy human subjects. The patients with atopic dermatitis are in the state of immune dysfunction characterized by decreased function of regulatory T cells and Th1 cells.^[34,35]^ Immune systems of healthy human subjects are in the homeostatic status (immune tolerance state). The difference in the baseline functional states of regulatory T cells might result in discrepancy of changes in serum IL-10 and IFN-γ levels between patients with atopic dermatitis and healthy human subjects. However, we cannot exclude a possibility that the dose and duration of intramuscular administration of autologous total IgG required to increase serum IL-10 and IFN-γ levels in healthy human subjects are higher and longer compared to patients with atopic dermatitis. Further studies are needed to explain the reason for the discrepancy of immunomodulatory effects induced by 8 intramuscular administrations of 50 mg autologous total IgG for 4 weeks between patients with atopic dermatitis and healthy human subjects.

In this study, the percentage of IL-10-producing CD4^+^ T cells increased significantly at week 8 and reached a peak at week 12 after intramuscular administrations of autologous total IgG in healthy human subjects. These results are consistent with the pattern of changes in serum levels of IL-10, which are also significantly increased at week 4, with the peak level reached at week 12 after the injections of the same amount of autologous total IgG for the same time period in adult patients with severe atopic dermatitis in our previous study.^[25]^ These results collectively suggest that T cell activation induced by intramuscular administration of autologous total IgG has characteristics of delayed onset and prolonged effect in human subjects. These immunomodulatory characteristics could be further supported by long-term clinical improvements and decrease in serum total IgE levels lasting for more than 9 months observed in 2 out of 3 adult patients with severe atopic dermatitis who were followed up for more than 2 years after 8 intramuscular administrations of 50 mg autologous total IgG for 4 weeks.^[24]^

It has been reported that the addition of polyvalent human IgG to purified CD4^+^ T cells from healthy human subjects increased intracellular expression of IL-10 in CD4^+^ CD25^high^ T cells, suggesting a direct activation of regulatory T cells by polyvalent IgG.^[8]^ Interestingly, the expansion of IL-10-producing CD4^+^ T cells induced by intramuscular administration of autologous total IgG in healthy human subjects observed in this study was similar to the expansion of IL-10-producing CD4^+^ T cells induced by intravenous administration of heterologous polyvalent human IgG in patients with autoimmune diseases, including immune thrombocytopenia and eosinophilic granulomatosis with polyangiitis.^[36,37]^ The doses of intravenously administered heterologous polyvalent human IgG that ameliorated autoimmune or inflammatory diseases and expanded regulatory T cells were high (1–2 g/kg body weight every 3 or 4 weeks).^[36–38]^ However, the dose of intramuscularly administered autologous total IgG required for clinical improvements in adult patients with severe atopic dermatitis^[16,23–25]^ and for the expansion of IL-10-producing CD4^+^ T cells in healthy adult human subjects observed in this study, was relatively low (total 400 mg; 8 injections of 50 mg autologous total IgG for 4 weeks). The reason for requirement of high dose of polyvalent human IgG to treat autoimmune or allergic diseases was attributed to low concentration and low affinity of natural anti-idiotype antibodies to pathogenic autoantibodies contained in polyvalent human IgG^[3]^ Clinical trials investigating high-dose intravenous polyvalent IgG therapy in adult patients with severe atopic dermatitis showed little clinical benefit.^[38]^ The nature of immune response provoked by a specific antigen was determined by the dose and route of administration of the antigen.^[39,40]^ These results collectively suggest that the differences in dose (high or low), route of administration (intravenous or intramuscular), and origin of IgG (heterologous or autologous) could result in different kinds of immunomodulatory effects (passive anti-idiotypic immune modulation or active anti-idiotypic immune modulation).^[26]^ We speculate that intramuscularly administered high concentration of purified autologous total IgG can induce immunomodulatory effects in healthy human subject due to differences in antigen dosage, purity, and type of antigen-presenting cells involved, compared to the naturally present autologous total IgG in blood circulation.^[26]^ Further studies are necessary to evaluate the detailed mechanisms of systemic immunomodulation induced by intravenous or intramuscular administration of heterologous or autologous human IgG.

The intravenous polyvalent human IgG therapy is expensive due to the safety issues and manufacturing process of immunoglobulin preparation from the plasma pool of multiple blood donors; furthermore, there is a serious concern about the shortage of supply of blood donation in the present and in the future.^[3,41]^ A simple affinity purification is required for the preparation of autologous total IgG from autologous plasma, with a minimal risk of transmission of infectious agents due to this procedure. The cost for preparation of autologous total IgG from autologous plasma is apparently lower than the manufacturing cost of heterologous polyvalent human IgG.^[41]^ Therefore, our study suggests that the intramuscular administration of autologous total IgG could be a simple, safe, and economical immunomodulation method, with a potential to replace the intravenous polyvalent human IgG therapy, for patients with various immune diseases.

The major limitation of our study is the absence of mechanistic insight into the T cell immunomodulation induced by intramuscular administration of autologous total IgG. We initially hypothesized that the intramuscular administration of autologous total IgG could induce a systemic immunomodulatory effect through the anti-idiotypic immunomodulation. Although we showed the induction of significant immunomodulatory effects on T cells, we could not provide any direct evidence of the development of anti-idiotype T cells or anti-idiotype antibody responses in this study. In the previous study, we attempted to measure the changes in serum levels of IgG anti-idiotype antibodies to autologous total IgG by ELISA in 3 adult patients with severe atopic dermatitis.^[24]^ However, we could not detect any significant changes in serum levels of IgG antibodies to the F(ab’)_2_ fragments of autologous total IgG in those patients before and after intramuscular administration of autologous total IgG.^[24]^

This study has several limitations as a clinical trial. This was an uncontrolled clinical trial, without a placebo control group, and included relatively small number of participants due to small amount of research grant. The intervention duration of the study was also relatively short (4 weeks). Further clinical trials with a randomized placebo-controlled study design, a larger number of subjects, and longer intervention duration are needed to confirm the immunomodulatory effects of intramuscular administration of autologous total IgG in healthy human subjects.

In conclusion, intramuscular administration of autologous total IgG induced immunomodulatory effects on T cells in healthy human subjects. This simple intervention could be a safe, effective, and economical T cell immunomodulation method for human subjects. Further studies are required to evaluate the detailed mechanism of this immunomodulatory effect.

## Author contributions

D-HN determined the study concept, participated in the study design, analysis and interpretation of the data, funding, and drafting of the manuscript, and has the final responsibility for the submission of the manuscript for publication. BK, S-JY, S-MC, and M-EK contributed to the acquiring of data. BK performed experiments and data extraction, and interpretation of flow cytometry assay. S-JY and S-MC contributed to the measurement and analysis of the serological changes. BK and M-EK contributed to the statistical analysis and interpretation of the data. S-JY and M-EK conducted the administrative, technical or material support, and management of this study. All authors contributed in the writing to the manuscript and approved the final version of the manuscript.

**Conceptualization:** Dong-Ho Nahm.

**Data curation:** Byul Kwon, Seung-Jung Yang, Su-Mi Cho, Myoung-Eun Kim, Dong-Ho Nahm.

**Formal analysis:** Byul Kwon, Seung-Jung Yang, Su-Mi Cho, Myoung-Eun Kim, Dong-Ho Nahm.

**Investigation:** Dong-Ho Nahm.

**Methodology:** Byul Kwon, Dong-Ho Nahm.

**Project administration:** Byul Kwon, Dong-Ho Nahm.

**Visualization:** Byul Kwon, Myoung-Eun Kim, Dong-Ho Nahm.

**Writing – original draft:** Byul Kwon, Seung-Jung Yang, Su-Mi Cho, Myoung-Eun Kim, Dong-Ho Nahm.

**Writing – review & editing:** Byul Kwon, Seung-Jung Yang, Su-Mi Cho, Myoung-Eun Kim, Dong-Ho Nahm.

## Supplementary Material

Supplemental Digital Content
