# Supplementary material for: Intramuscular administration of autologous total immunoglobulin G induces immunomodulatory effects on T cells in healthy human subjects: An open-labeled prospective single-arm trial
Source: Medicine (Baltimore). 2022 Jun 3;101(22):e29486. doi: 10.1097/MD.0000000000029486 (PMC9276166; doi:10.1097/MD.0000000000029486)
Supplement: Supplemental Digital Content [file medi-101-e29486-s001.doc]

**Supplemental Table 1.** The percentages of IL-10- or IFN-γ-producing peripheral blood CD3^+^ (a), CD4^+^ (b), CD8^+^ (c), CD25^+^ (d), and CD4^+^CD25^+^ (e) T cells in 13 healthy human subjects who received 8 intramuscular administration of 50 mg autologous total IgG twice a week for 4 weeks (from week 0 to week 4)

(a) Percentage of IL-10- or IFN-γ-producing cells in CD3^+^ T cells

| **CD3^+^ T cells** | | | | | | | | | |
| --- | --- | --- | --- | --- | --- | --- | --- | --- | --- |
| Subject  no. | IL-10^+^ cells in CD3^+^ T cells (%) | | | |  | IFN-r^+^ cells in CD3^+^ T cells (%) | | | |
|  | Baseline  (Week 0) | Week 4 | Week 8 | Week 12 |  | Baseline  (Week 0) | Week 4 | Week 8 | Week 12 |
| 1 | 1.6 | 1.8 | 1.3 | 0.9 |  | 34.6 | 35 | 33.5 | 33.8 |
| 2 | 0.5 | 1.2 | 0.7 | 1.8 |  | 25.1 | 26.8 | 17.4 | 26.3 |
| 3 | 0.4 | 0.7 | 0.5 | 1.1 |  | 16.8 | 35.4 | 31.9 | 32.8 |
| 4 | 0.7 | 1.6 | 2.3 | 2.3 |  | 30.0 | 27.0 | 36.0 | 37.2 |
| 5 | 1.8 | 1.5 | 0.9 | 2.4 |  | 18.9 | 16.8 | 20.0 | 22.5 |
| 6 | 0.7 | 0.9 | 1.0 | 0.9 |  | 27.7 | 29.3 | 27.1 | 28.6 |
| 7 | 0.7 | 0.7 | 0.7 | 1.1 |  | 28.4 | 21.9 | 24.0 | 24.4 |
| 8 | 0.5 | 1.6 | 1.1 | 0.5 |  | 35.4 | 42.5 | 34.3 | 39.6 |
| 9 | 0.5 | 1.8 | 1.1 | 0.9 |  | 13.8 | 31.5 | 25.6 | 23.1 |
| 10 | 0.7 | 1.7 | 0.5 | 1.1 |  | 8.7 | 21.8 | 20.2 | 18.2 |
| 11 | 0.4 | 0.3 | 0.2 | 1.2 |  | 30.4 | 25.9 | 34.3 | 30.3 |
| 12 | 0.2 | 0.8 | 0.4 | 0.6 |  | 22.9 | 27.6 | 24.0 | 24.9 |
| 13 | 0.2 | 0.5 | 0.1 | 0.5 |  | 13.5 | 15.4 | 16.1 | 18.9 |

(b) Percentage of IL-10- or IFN-γ-producing cells in CD4^+^ T cells

| **CD4^+^ T cells** | | | | | | | | | |
| --- | --- | --- | --- | --- | --- | --- | --- | --- | --- |
| Subject  no. | IL-10^+^ cells in CD4^+^ T cells (%) | | | |  | IFN-r^+^ cells in CD4^+^ T cells (%) | | | |
|  | Baseline  (Week 0) | Week 4 | Week 8 | Week 12 |  | Baseline  (Week 0) | Week 4 | Week 8 | Week 12 |
| 1 | 1.0 | 2.1 | 1.1 | 2.4 |  | 35.0 | 60.3 | 28.8 | 38.6 |
| 2 | 0.1 | 1.1 | 2 | 1.2 |  | 16.2 | 24.8 | 12.8 | 26.3 |
| 3 | 0.3 | 1.8 | 5.2 | 1.6 |  | 15.5 | 38.0 | 33.9 | 39.1 |
| 4 | 0.1 | 0.9 | 0.5 | 4.1 |  | 41.7 | 35.6 | 46.5 | 50.6 |
| 5 | 0.4 | 0.3 | 1.4 | 2.9 |  | 21.1 | 17.1 | 26.6 | 24.8 |
| 6 | 1.2 | 0.9 | 0.2 | 10.5 |  | 34.9 | 36.0 | 41.8 | 41.8 |
| 7 | 0.5 | 1.7 | 1.0 | 1.9 |  | 24.4 | 31.2 | 21.4 | 24.3 |
| 8 | 0.7 | 0.3 | 1.4 | 0.8 |  | 42.9 | 33.4 | 28.9 | 35.4 |
| 9 | 0.4 | 0.4 | 6.3 | 0.3 |  | 30.7 | 26.2 | 22.2 | 16.6 |
| 10 | 0.4 | 4.9 | 0.6 | 0.9 |  | 5.5 | 21.3 | 17.6 | 17.6 |
| 11 | 0.3 | 0.3 | 0.2 | 2.6 |  | 33.7 | 39.0 | 38.4 | 29.0 |
| 12 | 0.1 | 0.3 | 1.8 | 1.6 |  | 19.7 | 27.3 | 42.2 | 53.8 |
| 13 | 0.2 | 0.2 | 1.7 | 1.9 |  | 5.3 | 9.3 | 20.5 | 19.9 |

(c) Percentage of IL-10- or IFN-γ-producing cells in CD8^+^ T cells

| **CD8^+^ T cells** | | | | | | | | | |
| --- | --- | --- | --- | --- | --- | --- | --- | --- | --- |
| Subject  no. | IL-10^+^ cells in CD8^+^ T cells (%) | | | |  | IFN-r^+^ cells in CD8^+^ T cells (%) | | | |
|  | Baseline  (Week 0) | Week 4 | Week 8 | Week 12 |  | Baseline  (Week 0) | Week 4 | Week 8 | Week 12 |
| 1 | 0.4 | 1.3 | 0.3 | 0.2 |  | 57.6 | 63.6 | 57.8 | 59.7 |
| 2 | 0.6 | 1.3 | 0.2 | 0.8 |  | 35.6 | 39.8 | 23.4 | 37.4 |
| 3 | 0.2 | 0.3 | 0.6 | 0.4 |  | 27.4 | 40.1 | 42.6 | 44.1 |
| 4 | 0.3 | 0.3 | 0.7 | 0.1 |  | 56.1 | 58.0 | 59.8 | 68.6 |
| 5 | 0.4 | 0.1 | 0.1 | 0.3 |  | 24.1 | 19.3 | 20.7 | 25.9 |
| 6 | 0.6 | 0.4 | 0.2 | 0.4 |  | 68.8 | 76.5 | 70.3 | 71.8 |
| 7 | 0.6 | 0.1 | 0.2 | 0.6 |  | 41.1 | 34.8 | 34.6 | 34.7 |
| 8 | 0.2 | 0.4 | 0.1 | 0.1 |  | 51.6 | 62.5 | 50.7 | 54.0 |
| 9 | 0.1 | 0.1 | 0.5 | 0.3 |  | 25.8 | 42.5 | 32.5 | 24.0 |
| 10 | 0.2 | 0.7 | 0.3 | 0.4 |  | 11.6 | 25.9 | 24.0 | 24.9 |
| 11 | 0.1 | 0.3 | 0.1 | 0.5 |  | 46.8 | 35.0 | 39.2 | 40.4 |
| 12 | 0.1 | 0.2 | 0.2 | 0.3 |  | 46.2 | 61.7 | 47.6 | 46.4 |
| 13 | 0.2 | 0.1 | 0.2 | 0.3 |  | 49.6 | 43.9 | 30.5 | 38.1 |

(d) Percentage of IL-10- or IFN-γ-producing cells in CD25^+^ T cells

|  | **CD25^+^ T cells** | | | | | | | | |
| --- | --- | --- | --- | --- | --- | --- | --- | --- | --- |
| Subject  no. | IL-10^+^ cells in CD25^+^ T cells (%) | | | |  | IFN-r^+^ cells in CD25^+^ T cells (%) | | | |
|  | Baseline  (Week 0) | Week 4 | Week 8 | Week 12 |  | Baseline  (Week 0) | Week 4 | Week 8 | Week 12 |
| 1 | 8.8 | 2.4 | 1.4 | 1.9 |  | 16.7 | 21.4 | 15.6 | 4 |
| 2 | 1.2 | 2.0 | 0.7 | 2.8 |  | 16.5 | 7.1 | 3.5 | 8.8 |
| 3 | 0 | 1.8 | 5.5 | 2.1 |  | 10 | 10.3 | 1.2 | 18.3 |
| 4 | 0 | 3.0 | 2.0 | 11.4 |  | 4.5 | 7.5 | 13.4 | 10.6 |
| 5 | 1.6 | 0.7 | 2.8 | 12.4 |  | 3.1 | 2.5 | 9.5 | 7 |
| 6 | 1.4 | 2.9 | 2.8 | 18.9 |  | 5.4 | 8.5 | 26.8 | 21.1 |
| 7 | 0.3 | 1.7 | 1.4 | 2.4 |  | 5.7 | 16.3 | 4.6 | 10.8 |
| 8 | 1.8 | 13.8 | 2.7 | 0.7 |  | 10.9 | 13.5 | 14.7 | 5.7 |
| 9 | 0.6 | 1.0 | 15.3 | 1.3 |  | 10.4 | 4.1 | 8 | 10.5 |
| 10 | 0.4 | 18.4 | 0.6 | 0.4 |  | 5.3 | 7.8 | 4.6 | 8.2 |
| 11 | 0.9 | 0.9 | 2.8 | 4.8 |  | 8.1 | 11.6 | 17.8 | 12 |
| 12 | 0.7 | 1.5 | 2.4 | 3.0 |  | 5.3 | 8.6 | 6.7 | 5 |
| 13 | 4.0 | 1.5 | 1.6 | 1.3 |  | 6.8 | 5.3 | 6.8 | 7 |

(e) Percentage of IL-10- or IFN-γ-producing cells in CD4^+^CD25^+^ T cells

|  | **CD4^+^CD25^+^ T cells** | | | | | | | | |
| --- | --- | --- | --- | --- | --- | --- | --- | --- | --- |
| Subject  no. | IL-10^+^ cells in CD4^+^CD25^+^ T cells (%) | | | |  | IFN-r^+^ cells in CD4^+^CD25^+^ T cells (%) | | | |
|  | Baseline  (Week 0) | Week 4 | Week 8 | Week 12 |  | Baseline  (Week 0) | Week 4 | Week 8 | Week 12 |
| 1 | 0.2 | 0.2 | 0.1 | 0.2 |  | 0.6 | 0.8 | 0.5 | 0.2 |
| 2 | 0.1 | 0 | 0.3 | 0.2 |  | 0 | 0.4 | 0.2 | 0.2 |
| 3 | 0 | 0.1 | 0 | 0.2 |  | 0 | 0.4 | 0.3 | 0.2 |
| 4 | 0 | 0.3 | 3.6 | 0.2 |  | 0 | 0.1 | 0.3 | 0.2 |
| 5 | 2.3 | 0.1 | 0.3 | 0.3 |  | 0.1 | 0.2 | 0.3 | 0 |
| 6 | 0.2 | 0.2 | 0 | 0.1 |  | 0.1 | 0.1 | 0.5 | 0.2 |
| 7 | 0.1 | 0 | 0.4 | 0.1 |  | 0 | 0.9 | 0.3 | 0 |
| 8 | 0.1 | 0.2 | 0.4 | 0 |  | 0.4 | 0.3 | 0.1 | 0.5 |
| 9 | 0 | 0.5 | 1.2 | 0.4 |  | 1.8 | 0.5 | 0.2 | 0 |
| 10 | 0 | 0.1 | 0 | 0.1 |  | 0.1 | 0.1 | 0 | 0.2 |
| 11 | 0 | 0.5 | 0.1 | 0.1 |  | 0.4 | 0.8 | 0.4 | 0.4 |
| 12 | 0.1 | 0.1 | 0.2 | 0.4 |  | 0.2 | 0.4 | 0.5 | 0.2 |
| 13 | 0.1 | 0.1 | 0.2 | 0.4 |  | 0.4 | 0.2 | 0.4 | 0.4 |
